# Threonine^175^, a novel pathological phosphorylation site on tau protein linked to multiple tauopathies

**DOI:** 10.1186/s40478-016-0406-4

**Published:** 2017-01-11

**Authors:** Alexander J. Moszczynski, Wencheng Yang, Robert Hammond, Lee Cyn Ang, Michael J. Strong

**Affiliations:** 1 Molecular Medicine Research Group, Robarts Research Institute, 1151 Richmond Street North, London, N6A 5B7 ON Canada; 2 University Hospital LHSC, Rm A3-148, 339 Windermere Rd., London, ON N6A 5A5, Canada; 3 Molecular Medicine Research Group, Robarts Research Institute, 1151 Richmond Street North, London, N6A 5B7 ON Canada; 4 Department of Clinical Neurological Sciences, Schulich School of Medicine & Dentistry, Western University, London, ON Canada

**Keywords:** Microtubule associated protein tau, Tauopathy, Amyotrophic lateral sclerosis, Frontotemporal dementia, Alzheimer’s disease, Neuronal toxicity

## Abstract

Microtubule associated protein tau (tau) deposition is associated with a spectrum of neurodegenerative diseases collectively termed tauopathies. We have previously shown that amyotrophic lateral sclerosis (ALS) with cognitive impairment (ALSci) is associated with tau phosphorylation at Thr^175^ and that this leads to activation of GSK3β which then induces phosphorylation at tau Thr^231^. This latter step leads to dissociation of tau from microtubules and pathological tau fibril formation. To determine the extent to which this pathway is unique to ALS, we have investigated the expression of pThr^175^ tau and pThr^231^ tau across a range of frontotemporal degenerations. Representative sections from the superior frontal cortex, anterior cingulate cortex (ACC), amygdala, hippocampal formation, basal ganglia, and substantia nigra were selected from neuropathologically confirmed cases of Alzheimer’s disease (AD; *n* = 3), vascular dementia (*n* = 2), frontotemporal lobar degeneration (FTLD; *n* = 4), ALS (*n* = 5), ALSci (*n* = 6), Parkinson’s disease (PD; *n* = 5), corticobasal degeneration (CBD; *n* = 2), diffuse Lewy body dementia (DLBD; *n* = 2), mixed DLBD (*n* = 3), multisystem atrophy (MSA; *n* = 6) and Pick’s disease (*n* = 1) and three neuropathologically-normal control groups aged 50–60 (*n* = 6), 60–70 (*n* = 6) and 70–80 (*n* = 8). Sections were examined using a panel of phospho-tau antibodies (pSer^208,210^, pThr^217^, pThr^175^, pThr^231^, pSer^202^ and T22 (oligomeric tau)). Across diseases, phospho-tau load was most prominent in layers II/III of the entorhinal cortex, amygdala and hippocampus. This is in contrast to the preferential deposition of phospho-tau in the ACC and frontal cortex in ALSci. Controls showed pThr^175^ tau expression only in the 7^th^ decade of life and only in the presence of tau pathology and tau oligomers. With the exception of DLBD, we observed pThr^175^ co-localizing with pThr^231^ in the same cell populations as T22 positivity. This suggests that this pathway may be a common mechanism of toxicity across the tauopathies.

**Electronic supplementary material:**

The online version of this article (doi:10.1186/s40478-016-0406-4) contains supplementary material, which is available to authorized users.

## Introduction

Microtubule associated protein tau (tau) is a cytoskeletal stabilizing protein involved in microtubule maintenance, fast axonal transport, and other physiological functions in neurons. Tau protein deposition is a characteristic of many neurodegenerative diseases that are collectively referred to as tauopathies. It has been shown that pathological species of tau protein are abnormally phosphorylated at multiple residues [1] and that this is linked to a decrease in tau’s ability to bind to and stabilize microtubules [2, 3] with accompanying cytotoxicity [4]. While the isoform composition of insoluble tau deposits and the structural formation of the protein aggregates differs amongst the tauopathies, there are several phosphorylation sites that are thought to be universally important in the induction of a tauopathy.

One potentially important phosphorylation site that has gone relatively unstudied is threonine^175^ (Thr^175^). First identified in Alzheimer’s disease as a unique phosphoepitope [5], it was then determined that this site could be phosphorylated by multiple kinases linked to tau protein pathology, including GSK3β, JNK, ERK2, and p38 [6]. pThr^175^ tau was then identified in amyotrophic lateral sclerosis with cognitive impairment (ALSci) [7] and characterized in further detail in the context of this disease [8–12]. Importantly, pThr^175^ tau has been shown to induce tau fibril formation and cell death in vitro [13]. Unlike other widely accepted pathological phosphorylation sites on tau, such as pThr^231^ and pSer^202^, pThr^175^ has not been observed in the fetal brain where tau protein is hyperphosphorylated [14–16], suggesting that this site may be uniquely associated with pathological processes. pThr^175^ tau has been shown to induce GSK3β activation in cell culture and may therefore act as a destabilizing event resulting in enhanced phosphorylation of tau at other residues, resulting in dissociation from microtubules and neuronal toxicity [17]. In order to understand the extent to which this pathway of pThr^175^ mediated tau aggregate formation underlies a broad range of tauopathies, we have used a panel of phospho-specific antibodies to characterize tau protein pathology with specific interest in the expression of pThr^175^ tau across a broad range of tauopathies.

## Materials and methods

Diseases studied included Alzheimer’s (AD; 3 cases), vascular dementia (VD; 2 cases), ALS (5 cases), ALSci (6 cases), dementia with Lewy Bodies (DLBD; 2 cases), DLBD with mixed pathology (mDLBD; 3 cases including 2 with DLBD/VD and 1 with DLBD/AD)), frontotemporal lobar dementia (FTLD-TDP; 3 cases including one with a pathological C9orf72 hexanucleotide expansion with Type B pathology; a single case with Type A pathology and a single case with Type B pathology, FTLD-Tau; 1 case with familial history and no known mutations) [18], multiple system atrophy (MSA; 6 cases), Parkinson’s disease (PD; 5 cases), Pick’s disease (1 case), and corticobasal degeneration (CBD; 2 cases) (Table 1). The institutional research ethics board approved the protocol and consent was given for use of all tissue used in this study. All neuropathological diagnosis were performed by a neuropathologist (RH, LCA) and conformed to international neuropathological criteria [19–21]. For all comparisons, we grouped the staining according to ALS (*n* = 5), ALSci (*n* = 6), or other tauopathy (*n* = 22).

**Table 1:** Case demographics

Neuropathological diagnosis	Age	n (n Male)
AD	72 ± 8	3 (2)
VD	78 ± 11	2 (1)
ALS	56 ± 16	5 (4)
ALSci	64 ± 11	6 (5)
DLBD	68 ± 1	2 (2)
mDLDB	83 ± 6	3 (3)
FTLD	64 ± 9	4 (1)
MSA	69 ± 12	6 (3)
PD	77 ± 2	5 (4)
Pick’s	70 ± 2	2 (2)
CBD	71 ± 1	2 (1)
Control 1	55 ± 2	6 (4)
Control 2	64 ± 2	6 (4)
Control 3	75 ± 3	8 (4)

*AD* Alzheimer’s disease, *VD* Vascular dementia, *ALS* amyotrophic lateral sclerosis, *ALSci* ALS with cognitive impairment, *DLBD* diffuse Lewy body dementia, *mDLBD* Lewy body dementia with mixed pathology, *FTLD* frontotemporal lobar dementia, *MSA* multiple system atrophy, *PD* Parkinson’s disease, *Pick’s* Pick’s disease, *CBD* corticobasal degeneration. Control 1: 6^th^ decade control group, Control 2: 7^th^ decade control group, Control 3: 8^th^ decade control group

To assess the extent of pThr^175^ tau, pThr^231^ tau and tau oligomer pathological inclusions as a function of ageing, three groups of controls were studied, encompassing the 6^th^ (*n* = 6), 7^th^ (*n* = 6), and 8^th^ (*n* = 8) decades of life (Table 1). Hippocampal sections from each group were stained for pThr^175^ tau, pThr^231^ tau and oligomeric tau (T22). These cases have been previously characterized in a study examining age-dependant tau deposition in the frontal and entorhinal cortices and were shown to be free of neurodegenerative disease [22].

Five to six micrometer paraffin-embedded sections from the superior frontal gyrus, anterior cingulate (ACC), hippocampus, entorhinal cortex, dentate gyrus, amygdala, basal ganglia and substantia nigra were used for all immunohistochemical analyses.

Cases were stained by haematoxylin and eosin (H&E) and Gallyas silver stain for routine histological analysis and overall pathology characterization. Immunohistochemistry was conducted using a series of antibodies (Table 2) previously characterized in ALSci [12], consisting of PHF tau (AT8; Thermo Fischer IL, Canada), pThr^175^ tau, pSer^208,210^ tau, pThr^217^ tau (antibodies generated and designed in house [12], pThr^175^ commercially available through 21^st^ Century, MA, USA).

**Table 2:** Antibodies

Antibody	Clone	Titre	Antigen retrieval	Epitope	Company
Tau pThr^175^	Rabbit, polyclonal	1:1000	1	pThr^175^	21^st^ Century
Tau pThr^217^	Rabbit, polyclonal	1:1000	1	pThr^217^	21^st^ Century
Tau pSer^208, 210^	Rabbit, polyclonal	1:1000	1	pSer^208, 210^	21^st^ Century
PHF (AT8)	Mouse, monoclonal	2.5 ug/ml	No	pSer^202^	Thermo Fischer
Tau pThr^231^	Rabbit, polyclonal	1:1000	2	pThr^231^	Thermo Fischer
T22	Rabbit, polyclonal	1:500	2	Tau oligomer	EMD Millipore
Alexa Fluor 488	Goat anti-rabbit	1:200	2	Secondary	Life Technologies

1) Boil in 10 mM sodium citrate, 0.05% Tween 20 pH 6.0 for 2 min

2) Pressure cooker (2100 Retriever; Aptum Biologics, UK) 10 mM sodium citrate, 0.05% Tween 20 pH 6.0 for 15 min

Antigen retrieval was conducted as necessary (Table 2). Endogenous peroxidase was quenched with 3% hydrogen peroxide (BDH Chemicals, VWR, On, Canada). Primary antibody incubation was performed at 4 °C overnight in blocking buffer (5% BSA, 0.3% Triton-X 100 in 1 X PBS). After washing, secondary antibody (1:200 biotinylated IgG) incubation was performed for 1 h at room temperature in blocking buffer. Antigen:antibody complex was visualized with either horseradish peroxidase or alkaline phosphatase according to the manufacturer’s instructions (Vectastain ABC kit, Vector Laboratories CA, USA), followed by substrate development with either DAB plus NiCl_2_ or AP substrate kit III (Vector Laboratories). Counterstaining was performed using haematoxylin or nuclear fast red. The extent of pathology was described topographically and semi-quantitatively as previously reported [12]. Representative images were captured with a 20x lens under light microscopy (Olympus BX45) and subsequently used for semi-quantitative analysis. The semi-quantitative scale was manually applied for each type of pathology by an evaluator blinded to the underlying diagnosis (WY) (neuronal, neuritic, or glial) separately as follows: ‘-’ = none; ‘±’ = less than 5 inclusions; ‘+’ = less than 10 inclusions; ‘++’ = more than 20 inclusions with scattered distribution; ‘+++’ = more than 20 inclusions but with locally dense distribution; ‘++++’ = more than 20 inclusions with a diffuse distribution. Additionally, the case positive ratio was defined for each antibody used and brain region investigated as the number of cases showing any pathology (± or more) compared to the total number of cases stained.

### Oligomeric tau and pThr^231^ staining

Rabbit anti T22 (EMD Millipore CA, USA) and rabbit anti tau pThr^231^ (Thermo Fischer) were used to probe tau inclusions for the presence of oligomeric tau (T22) and for phosphorylation at Thr^231^. Tau oligomeric species are currently hypothesized to be more toxic to neurons than the fibrillar inclusions themselves [23], and pThr^231^ is thought to be a key site in the regulation of tau protein folding and ability to interact with microtubules [3, 24]. Double labeling was performed on hippocampus from one case each from AD, ALSci, FTD, MSA, DLDB, and mDLDB. Tau protein was probed for pThr^175^ using rabbit primary antibody (1:1000) overnight at 4 °C and Alexa Fluor goat anti-rabbit 488 nm secondary (1:200, Thermo Fischer) for 1 h at room temperature. Rabbit anti tau pThr^231^ antibody was then labeled using a Zenon primary antibody labeling kit with Alexa Fluor 555 nm dye (Thermo Fisher) and probed for 1 h at room temperature. Slides were stored overnight at 4 °C and visualized within 24 h of labeling by confocal imaging on a Zeiss LSM 510 Meta NLO multiphoton confocal microscope.

## Results

### Tau antibody staining

#### Neuronal tau

##### ALS

**Fig. 1:**
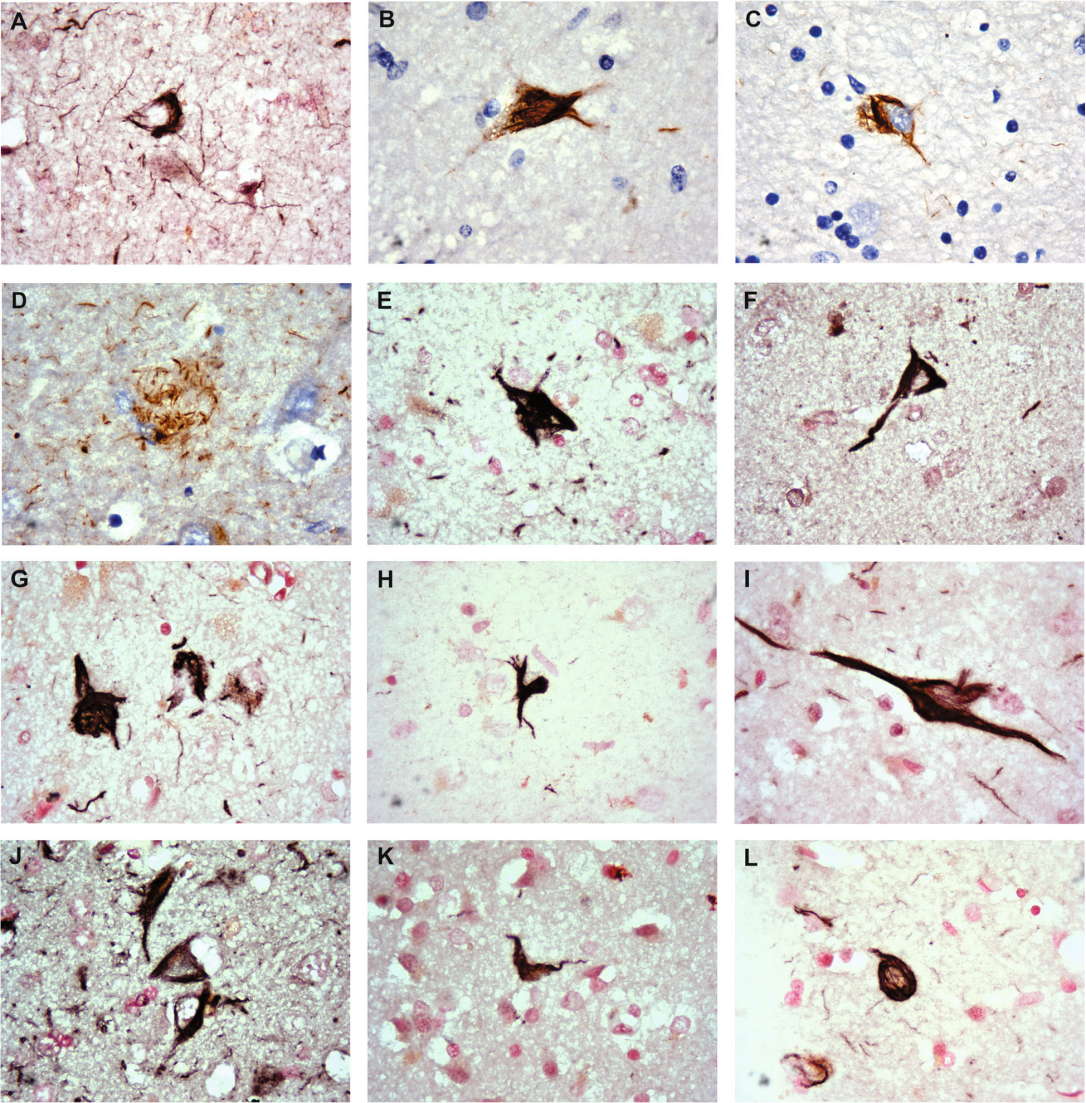
Representative pThr^175^ tau pathology in each neurodegenerative disease. **a** AD Frontal cortex, **b** ALS amygdala, **c** ALSci amygdala, **d** ALSci hippocampus neuritic plaque, **e** CBD entorhinal cortex, **f** DLBD amygdala, **g** mDLBD entorhinal cortex, **h** FTLD putamen, **i** MSA amygdala, **j** PD amygdala, **k** Pick’s entorhinal cortex, **l** VD anterior cingulate cortex. Nuclear fast red or hematoxylin counterstain used. Original images taken at 100×

Consistent with our earlier reports, we observed tau pathology in multiple brain regions in ALS, although to a lesser degree than either ALSci or the remaining tauopathies. Neuronal tau inclusions were most consistently observed in the entorhinal cortex, hippocampus, and amygdala. All antibodies were immunoreactive with neuronal tau inclusions in multiple brain regions (Table 3). Frontal and anterior cingulate pathology was limited in both load and case-positive incidence. In all regions studied, inclusions took the form of punctate cytosolic inclusions or tangles (Fig. 1). Deposition was mainly restricted to the superficial cortical layers in the entorhinal cortex but restricted to deeper layers in the ACC and superior frontal cortex when present.

**Table 3:** ALS pathology

Stain	Frontal	Cingulate	Hippocampus	Dentate	Entorhinal	Amygdala	BG	SN
Neuronal								
pThr^175^	± (1/5)	± (1/4)	± (1/5)	± (1/5)	± (2/4)	++ (1/2)	- (0/5)	- (0/5)
PHF	± (1/5)	± (1/4)	± (3/5)	± (2/5)	± − ++ (5/5)	+++ (1/1)	± (1/4)	± (3/4)
pSer^208,210^	- (0/5)	++ (1/4)	± (1/5)	- (0/4)	± (1/2)	++ (1/2)	- (0/5)	- (0/5)
pThr^217^	+ (1/5)	± (1/4)	± (1/5)	- (0/4)	± − ++ (3/4)	++ (1/2)	± (1/4)	± (2/5)
Glial								
pThr^175^	++ (1/5)	- (0/5)	- (0/4)	- (0/5)	- (0/2)	± (1/2)	+ (1/5)	- (0/5)
PHF	± (1/5)	± (1/4)	± (1/5)	- (0/5)	+ (1/5)	++ (1/1)	- (0/4)	- (0/4)
pSer^208,210^	- (0/5)	- (0/4)	- (0/5)	- (0/4)	± (1/2)	++ (1/2)	++ (2/5)	- (0/5)
pThr^217^	+++ (1/5)	+++ (1/4)	+ (1/5)	- (0/4)	+++ (1/4)	++ (1/2)	++ (2/4)	- (0/5)
Neuritic								
pThr^175^	- (0/5)	- (0/5)	± (1/4)	- (0/4)	± (2/5)	++ (1/2)	- (0/5)	± (1/5)
PHF	± (2/5)	± (1/4)	± (3/5)	- (0/5)	+ − ++ (5/5)	++ (1/1)	± (1/4)	± (3/4)
pSer^208,210^	- (0/5)	± (1/4)	- (0/5)	- (0/4)	± (1/2)	- (0/2)	+ (1/5)	+ (2/5)
pThr^217^	± (1/5)	- (0/4)	± (1/5)	- (0/4)	± − ++ (3/4)	± (1/2)	+ − ++ (2/4)	+ − ++ (3/5)

##### ALSci

Tau pathology (Fig. 1, Additional file 1: Figure S1, Additional file 2: Figure S2, Additional file 3: Figure S3) in the form of tangles, skein-like inclusions, and punctate staining was observed to a greater extent in ALSci than ALS, especially in the ACC and superior frontal cortex. The load of pathology was increased in amount and distribution and the case positive ratio was higher than ALS in all brain regions studied (Table 4). As observed in ALS, pathological tau neuronal inclusions were observed predominantly in the superficial layers of the entorhinal cortex and within deeper cortical layers in the ACC and superior frontal cortex. However superficial layer involvement was noted in both the ACC and superior frontal cortex in ALSci, indicating a greater distribution across cortical layers in ACC and frontal cortex, further differentiating ALSci from ALS. Of note, Thr^175^ tau and PHF tau identified pathology to different extents in different brain regions. Notably, pThr^175^ tau and pThr^217^ tau identified a higher case positive ratio than PHF in the superior frontal cortex.

**Table 4:** ALSci pathology

Stain	Frontal	Cingulate	Hippocampus	Dentate	Entorhinal	Amygdala	BG	SN
Neuronal								
pThr^175^	± (4/5)	± (2/5)	± (5/5)	+ (1/5)	+ (4/4)	+ (1/1)	- (0/5)	- (0/3)
PHF	+ (2/4)	± (3/3)	± − + (4/4)	± − + (2/4)	± − ++ (4/4)	± − ++ (2/2)	- (0/5)	± (2/2)
pSer^208,210^	± (2/5)	± (3/5)	± − ++ (4/5)	± (1/5)	± − ++ (3/4)	+ (1/1)	- (0/5)	- (0/3)
pThr^217^	± (5/5)	± − + (3/5)	± − ++ (5/5)	± − ++ (3/5)	± − ++ (5/5)	+ (1/1)	- (0/5)	± − + (2/2)
Glial								
pThr^175^	± − ++ (2/5)	± − ++ (2/5)	- (0/5)	- (0/5)	± (1/4)	- (0/1)	± − + (2/5)	- (0/3)
PHF	± − ++ (4/4)	± − ++ (2/3)	- (0/4)	- (0/4)	- (0/4)	+ (1/2)	± (3/5)	- (0/2)
pSer^208,210^	++ (4/5)	++ (2/5)	- (0/5)	- (0/5)	± (1/4)	- (0/1)	± − + (4/5)	± (1/3)
pThr^217^	++ − +++ (5/5)	+++ (2/5)	++ (2/5)	- (0/5)	± (1/5)	- (0/1)	+ − ++ (5/5)	- (0/2)
Neuritic								
pThr^175^	± − + (3/5)	± − + (2/5)	± − + (3/5)	± − ++ (4/5)	± − ++ (4/4)	- (0/1)	± (4/5)	± (1/3)
PHF	± − + (2/4)	± − + (3/3)	± − + (3/4)	± (3/4)	± − ++ (4/4)	± − ++ (2/2)	± (2/5)	± (2/2)
pSer^208,210^	± − + (2/5)	± (3/5)	± (4/5)	± (2/5)	± − ++ (4/5)	+ (1/1)	± (1/5)	± (1/3)
pThr^217^	± − ++ (5/5)	± − ++ (2/5)	± − + (4/5)	++ (2/5)	+ − +++ (5/5)	+ (1/1)	± (3/5)	± − ++ (2/2)

##### Tauopathies

Within the tauopathies and consistent with the literature, we observed tau neuronal pathology across multiple regions (Table 5). pThr^175^ tau was present in all disease states where tau pathology was prominent. In Alzheimer’s disease (AD), all tau antibodies showed robust neuronal pathology as neurofibrillary tangles and punctate cytoplasmic inclusions in all brain regions studied. Across all cortical regions studied, neuronal pathology was present across all cortical layers but was most prominent in deeper layers (IV-VI). Amongst the tauopathies, the most prominent pThr^175^ tau immunostaining was observed in AD. This included more prominent expression of pThr^175^ tau than observed in ALSci.

**Table 5:** Tauopathies pathology

Stain	Frontal	Cingulate	Hippocampus	Dentate	Entorhinal	Amygdala	BG	SN
Neuronal								
pThr^175^	± − ++ (10/27)	± − ++ (10/25)	± − +++ (20/26)	± − ++ (8/28)	± − +++ (21/28)	± − +++ (19/23)	± − + (10/29)	± (3/23)
PHF	± − ++++ (13/27)	± − +++ (16/24)	± − ++++ (28/29)	± − ++++ (18/29)	± − ++++ (28/29)	± − ++++ (21/21)	± − ++ (13/28)	± − ++ (7/23)
pSer^208,210^	± − +++ (12/27)	± − +++ (12/25)	± − +++ (25/29)	± − ++ (10/28)	± − +++ (23/28)	± − +++ (23/28)	± − ++ (9/28)	± − + (5/23)
pThr^217^	± − +++ (15/27)	± − +++ (14/24)	± − +++ (23/29)	± − ++ (12/29)	± − +++ (24/29)	± − +++ (19/21)	± − + (6/27)	± (7/23)
Glial								
pThr^175^	± − +++ (6/27)	± − ++ (6/25)	± − + (4/29)	± (1/28)	± (2/28)	± − ++ (3/24)	± − ++ (16/29)	± (3/23)
PHF	± − ++++ (8/27)	± − +++ (7/24)	± − + (5/29)	- (0/29)	± − +++ (8/29)	± − +++ (12/21)	± − ++++ (11/28)	- (0/23)
pSer^208,210^	+ − ++ (2/28)	± − +++ (3/25)	++ (3/29)	++ (1/28)	± − ++ (3/27)	± − +++ (3/22)	± − ++ (10/28)	- (0/23)
pThr^217^	± − +++ (7/26)	± − +++ (7/24)	± − +++ (3/29)	- (0/29)	++ − +++ (2/29)	± − +++ (4/21)	± − +++ (15/27)	- (0/23)
Neuritic								
pThr^175^	± − ++ (10/27)	± − ++ (9/25)	± − +++ (20/28)	± − ++ (6/28)	± − +++ (21/28)	± − +++ (16/24)	± − + (12/28)	± − ++ (10/23)
PHF	± − ++++ (14/27)	± − ++++ (15/24)	± − ++++ (24/29)	± − + (11/29)	± − ++++ (28/29)	± − ++++ (17/21)	± − +++ (16/28)	± − ++++ (13/23)
pSer^208,210^	± − ++++ (18/27)	± − +++ (9/25)	± − ++ (18/29)	± − + (6/28)	± − +++ (22/28)	± − +++ (18/23)	± − ++ (11/28)	± − ++ (11/23)
pThr^217^	± − ++++ (15/27)	± − ++++ (14/25)	± − ++ (21/29)	± − + (11/29)	± − +++ (24/29)	± − ++++ (18/23)	± − +++ (11/27)	± − ++ (11/23)

As in AD, VD exhibited tau protein deposition as tangles and punctate cytoplasmic inclusions in all brain regions studied. This followed the same trend as AD with pathology being most prominent in deep cortical layers. In CBD, balloon neurons were observed and tau pathology was prominent in all brain regions as punctate inclusions and neurofibrillary tangles. Notably, PHF tau staining was more intense than pThr^175^ tau in all regions, both in case positive ratio and in semiquantitative pathological load. In both DLBD and mDLBD, a similar degree of tau pathology was observed in the form of cytoplasmic punctate deposition and neurofibrillary tangles. Pathology within the dentate gyrus, basal ganglia and substantia nigra was present to a much greater extent in mDLBD than DLBD. In FTLD, tau pathology was observed as punctate inclusions and tangles in all brain regions investigated. In general, pThr^175^ tau was less prominent than PHF tau, except in the frontal and cingulate cortex where it was more prominent on a case positive basis and the pathological load observed. In MSA, tau pathology in the form of tangles and punctate inclusions was present in all brain regions studied although frontal and ACC pathology was sparse. In Parkinson’s disease, tau pathology was observed in all brain regions except the substantia nigra. Pathological tau expression was equivalent across all antibodies. In Pick’s disease, all brain regions investigated exhibited tau pathology in the form of tangles, punctate inclusions and Pick bodies. Notably, PHF tau pathology was greater than the other antibodies including pThr^175^ tau.

#### Neuritic tau

##### ALS

No neuritic plaques were observed in ALS. Neuritic pathology in the form of dystrophic neurites was observed to a limited extent in all brain regions studied and with a pattern of distribution mimicking that described above for neuronal pathology. Basal ganglia neuritic pathology was observed to a larger extent in the putamen than the globus pallidus by all tau antibodies but pThr^175^ tau. Neuritic tau pathology within the substantia nigra was immunoreactive against all antibodies employed in the analysis.

##### ALSci

Neuritic tau pathology was observed predominantly as dystrophic neurites assuming a short curvilinear morphology. This was consistently observed in both cortical and subcortical tissues. Contrary to the superficial localization of frontal and ACC neuronal pathology, frontal neuritic pathology was observed mainly in deep layers as short curved neurites. Entorhinal neuritic pathology was observed mainly in the superficial layers in proximity to tau inclusion bearing neurons. Neuritic plaques were observed in the entorhinal cortex by all antibodies with the exception of the pThr^175^ tau antibody. Neuritic plaques within the amygdala were observed by PHF tau antibody labeling only. Neuritic plaques and tau positive neurites were observed in the hippocampus and were immunoreactive to all antibodies. Coiled bodies were observed throughout the basal ganglia.

##### Tauopathies

Neuritic pathology was prominent across all the tauopathies and typically mirrored the presence of tau immunoreactive neuronal pathology. Neuritic plaques were observed in AD as small atypical plaques and typical plaques in deeper layers (IV/V) more frequently than in superficial layers (II/III). No antibody identified neuritic plaques in basal ganglia or substantia nigra. Like neuronal tau, all cortical neuritic pathology was most prominent in deeper layers (IV-VI). All antibodies recognized neuritic pathology, most commonly in deeper cortical layers near tau positive neurons as short and curved, or long, straight neurites. Neuritic pathology was also observed to a lesser extent in white matter in frontal, ACC, and entorhinal cortices.

Similar to AD, neuritic pathology in VD was present as tau positive neurites and neuritic plaques in all brain regions studied except the substantia nigra. Also similar to AD, neuritic pathology followed a tendancy to be most pronounced in regions of prominent neuronal tau pathology and in particular in the deeper cortical layers. In CBD, neuritic plaques were only observed in the ACC and substantia nigra, and then, only using the PHF tau antibody. Neuritic pathology, as dystrophic neurites, was present in all brain regions investigated and mirrored the distribution of neuronal pathology. This was most evident in the entorhinal cortices where dystrophic neurites were most evident in superficial cortical layers. In contrast, dystrophic neurites were most prominent in deep subcortical regions of the superior frontal cortex and ACC.

In DLBD, no frontal, ACC, dentate gyrus or basal ganglia neuritic pathology was observed. All other regions studied were positive for neuritic tau pathology, but no plaques were observed. Entorhinal neuritic pathology was most prominent in DLDB in layers II/III. In mDLBD, neuritic plaques were observed in all regions except for the substantia nigra. All regions studied exhibited neuritic pathology. In both FTLD and MSA, neuritic plaque pathology was not frequent and usually observed by PHF tau only. pThr^175^ tau neuritic pathology was not as prominent as that observed using other tau antibodies. However, neuritic tau pathology was observed in all brain regions studied as with neuronal pathology. In PD, neuritic plaques were not observed, but tau positive neurites were observed in a similar distribution to neuronal pathology in all brain regions studied. In Pick’s disease, neuritic pathology was identified mainly by PHF tau in all but the dentate gyrus.

#### Glial tau

##### ALS

Glial tau pathology was observed in all brain regions except the dentate gyrus, and substantia nigra (Table 3). Where present, glial pathology presented as glial fibrillary tangles and astrocytic plaques as previously described [12]. The distribution was sparse and followed that of neuronal and neuritic tau as described above and was similar in case positive ratio, although higher in semiquantitative load than neuronal and neuritic pathology in a single case.

##### ALSci

Glial pathology was present in ALSci (Fig. 1, Additional file 1: Figure S1, Additional file 2: Figure S2, Additional file 3: Figure S3) to a similar degree as in ALS, but was more frequent in terms of regional distribution, case positive rate, and pathological load (Table 4). Frontal and ACC glial pathology was increased in both case positive incidence and pathological load.

##### Tauopathies

In the tauopathies, the extent of glial pathology was highly dependant on the underlying disease (Table 5). In AD, glial pathology was rare, and when present was usually observed only in the amygdala and basal ganglia. No glial pathology was observed in VD. Consistent with the literature, CBD contained astrocytic plaques throughout the grey and white matter across multiple brain regions. Additional astrocytic staining and tau positive microglia were observed. Glial pathology in CBD was identified to a far greater extent by PHF tau than the pThr^175^ tau antibody. In DLBD, minimal glial pathology was observed in frontal cortex and basal ganglia, and when present, only as punctate astrocytic inclusions. In contrast to the limited glial pathology observed in DLBD, mDLBD showed much more frequent glial pathology across multiple brain regions, pathology that was less evident with the pThr^175^ tau antibody. In FTLD, glial pathology was observed in all brain regions except the dentate gyrus and substantia nigra. Notable in this disease, pThr^175^ tau identified glial pathology to a greater extent than PHF tau by both case positive incidence and increased load. In MSA, glial pathology was largely absent, being present only in the entorhinal cortex, amygdala and basal ganglia by multiple antibodies. Interestingly, the basal ganglia contained astrocytic inclusions in the putamen identified by all antibodies whereas in the globus pallidus they were only identified by pThr^175^ tau. In Parkinson’s disease, glial pathology was present in all regions except the dentate gyrus and substantia nigra. Notably, the PHF tau antibody identified astrocytic plaques in the frontal cortex while tufted astrocytes were observed in the amygdala. In Pick’s disease, glial pathology was observed in multiple brain regions using the PHF antibody mainly, although in the entorhinal cortex pThr^175^ tau was also positive for glial pathology.

#### pThr^231^ tau and T22 staining

**Fig. 2:**
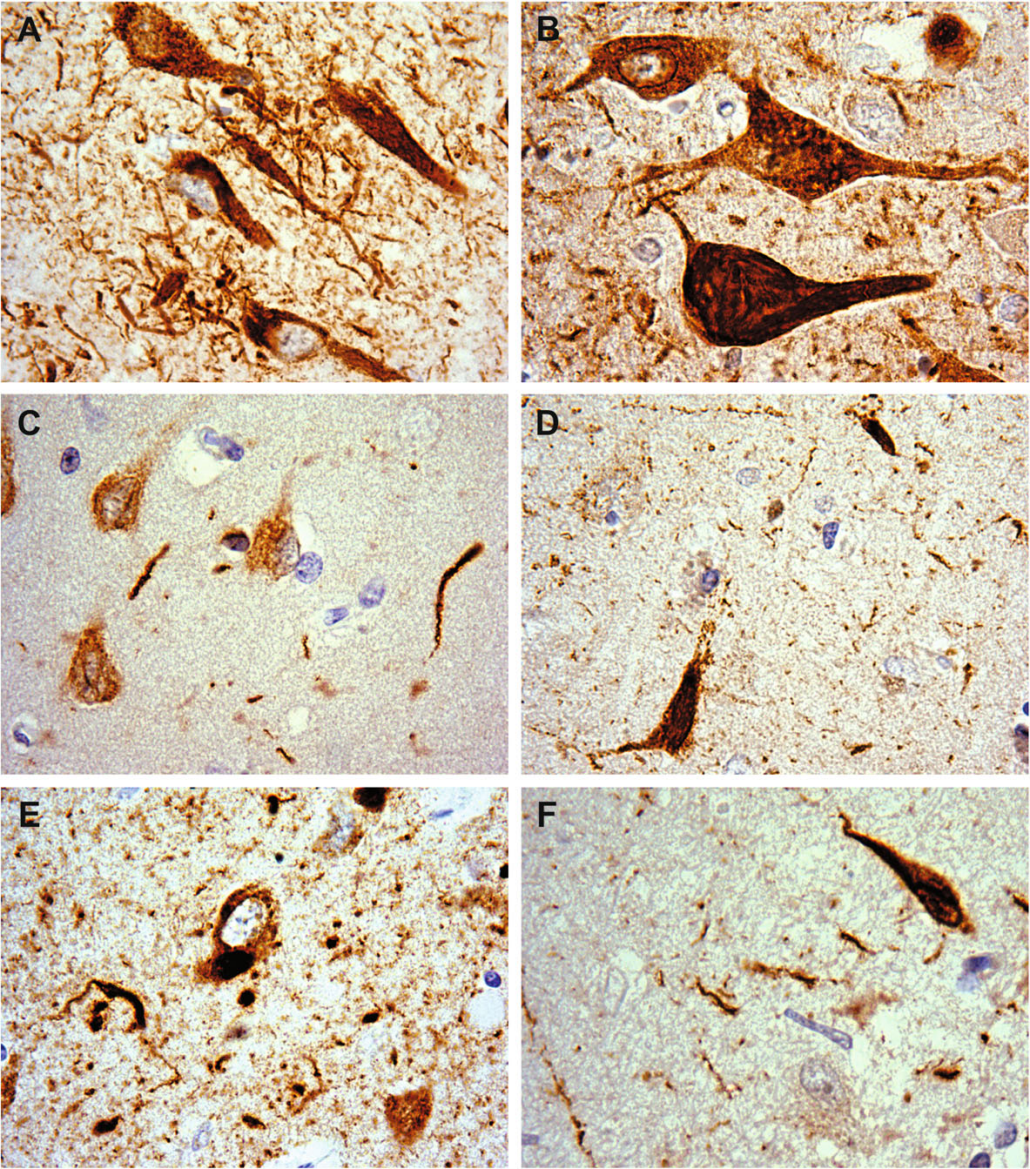
Representative hippocampal pThr^231^ tau pathology. **a** AD, **b** ALSci, **c** DLBD, **d** mDLBD, **e** FTLD, **f** MSA. Counterstained with hematoxylin. Original images taken at 100×

**Fig. 3:**
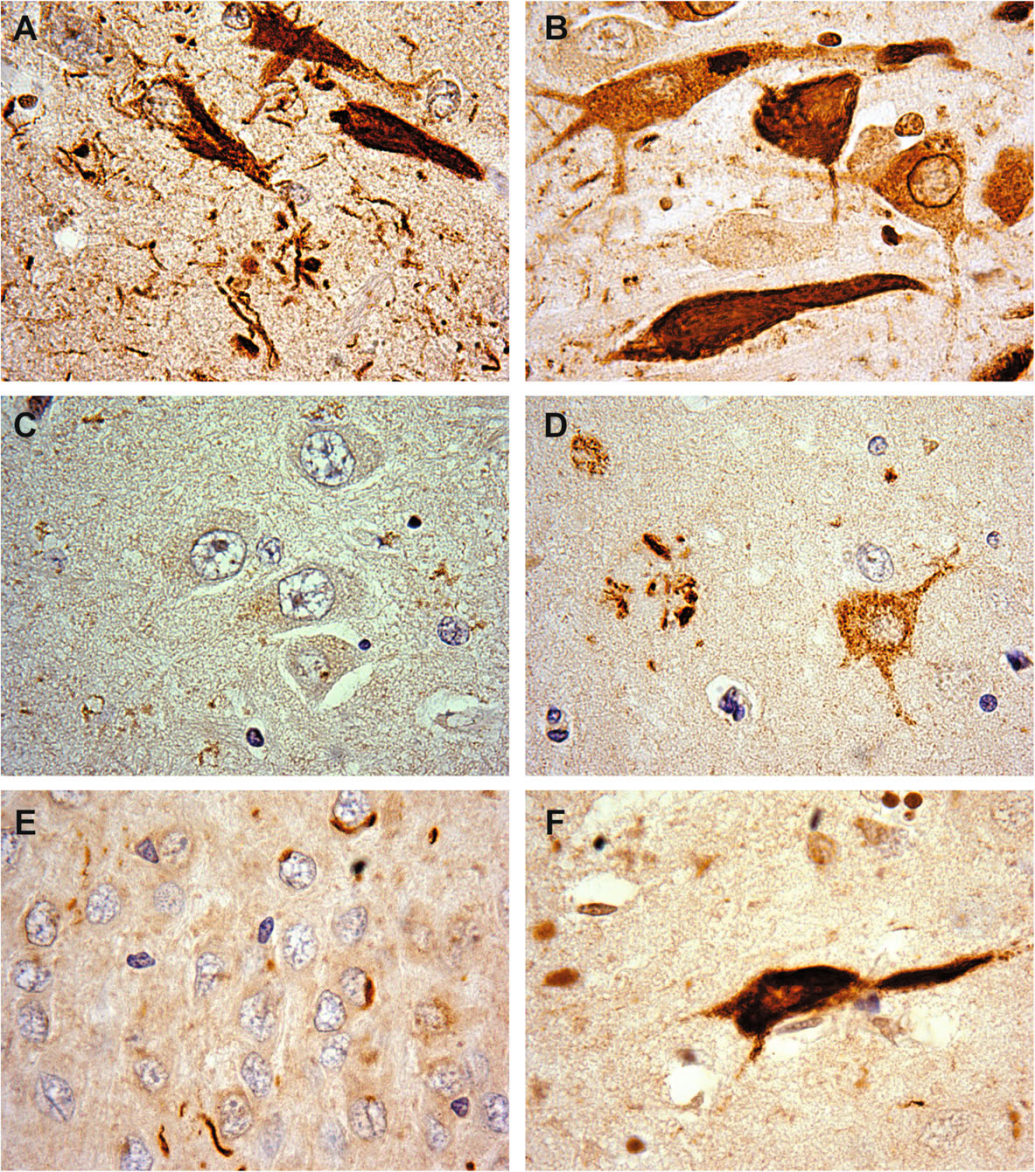
Representative hippocampal tau oligomer (T22) pathology. **a** AD, **b** ALSci, **c** DLBD, **d** mDLBD (**e**) FTLD (**f**) MSA. Counterstained with hematoxylin. Original images taken at 100×

We examined the presence of oligomeric tau (recognized by the T22 antibody) and pathological tau phosphorylation at Thr^231^ using sections from the hippocampus of a single case each of AD, ALSci, FTD, MSA, DLBD, and mDLB. Cases were selected on the basis of the pathology described earlier. In each case, tau neuronal inclusions were recognized by both antibodies (Figs. 2 and 3). Only DLBD showed notably reduced T22 pathology which, when present, was in the dystrophic neurites (Fig. 3). Glial pathology was not observed with either antibody, regardless of diagnosis.

AD neuronal pathology was observed as fibrillar and punctate inclusions by pThr^231^ tau antibody but only as fibrillar/tangles with T22. Neuritic pathology in the form of short and long torsional and dystrophic neurites was observed. pThr^231^ tau, but not T22, recognized plaques consisting of dystrophic neurites.

Similar to AD, ALSci neuronal pathology was observed by pThr^231^ tau and T22 as fibrillar and punctate staining. However additional solitary cytoplasmic inclusions were observed in neurons with the T22 antibody. Neuritic pathology was observed using both the pThr^231^ tau and T22 antibodies. Neuritic plaques were identified by both the pThr^231^ tau and T22 antibodies.

FTLD pathology was distinct from AD and ALSci in that pThr^231^ tau neuronal pathology was observed as a dense nuclear ring staining around abnormally folded nuclei, and solitary cytosolic inclusions on homogenously stained cytosol. While T22 neuronal cytoplasmic pathology was observed, neuritic pathology was observed as frequent short dystrophic neurites by both antibodies. No neuritic plaques were observed.

MSA pathology resembled AD and ALSci. Neuronal pathology was observed as tangles and punctate inclusions by both the pThr^231^ tau and T22 antibodies. In addition, pThr^231^ tau antibody diffuse cytoplasmic immunostaining was observed. Neuritic pathology was observed by both antibodies, although T22 immunoreactive neurites demonstrated a punctate staining pattern.

DLBD tau pathology was observed only faintly by pThr^231^ tau as punctate cytosolic deposition. While neuritic pathology was observed using the pThr^231^ tau antibody, no pathology was observed by T22 other than a few sporadic neurites. Conversely, mDLBD pathology was observed by both pThr^231^ tau and T22. pThr^231^ tau pathology was observed as tangles and punctate staining accompanied by dystrophic neurites and other neuritic pathology. No neuritic plaques were observed. T22 pathology however was observed as punctate cytosolic staining and dystrophic neurites.

**Fig. 4:**
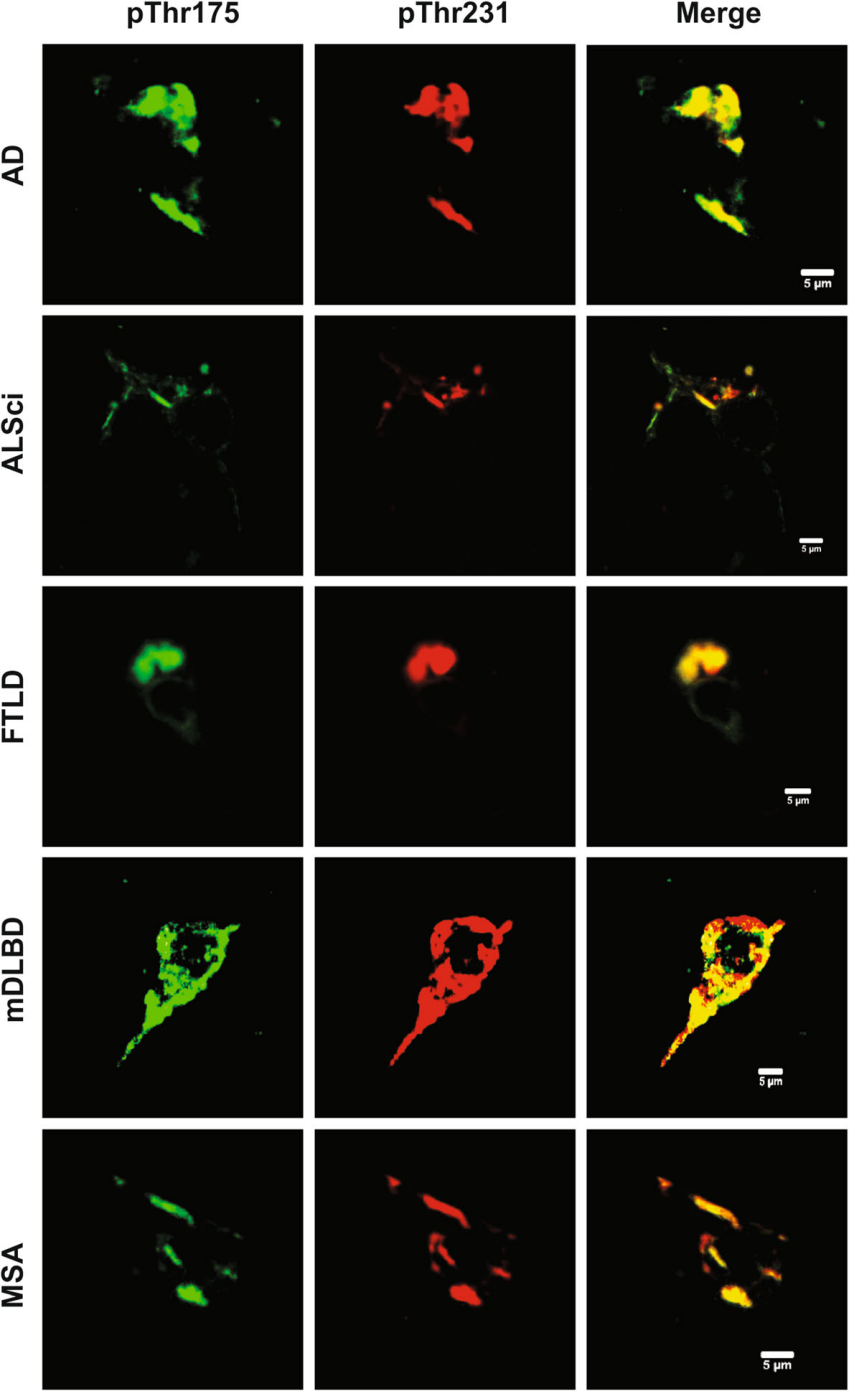
Co-localization of pThr^175^ and pThr^231^ tau in hippocampal neuronal inclusions. AD: Alzheimer’s disease. ALSci: amyotrophic lateral sclerosis with cognitive impairment. FTLD: frontotemporal lobar dementia. mDLBD: mixed diffuse dementia with Lewy bodies. MSA: Multiple system atrophy. Scale bar represents 5 μm

Having confirmed that both T22 and pThr^231^ tau immunoreactive pathology was present, although as described to varying degrees, we next sought to confirm whether pThr^175^ tau and pThr^231^ tau co-localized using confocal imaging (Fig. 4). Colocalization within neuronal tau inclusions was observed between pThr^175^ tau and pThr^231^ tau in each disease state except for DLBD. Colocalization was also observed in neuritic plaques in AD. No pThr^175^ tau immunoreactivity was observed in the absence of pThr^231^.

#### Hippocampal pThr^175^, pThr^231^ and oligomeric tau deposition as a function of aging

**Fig. 5:**
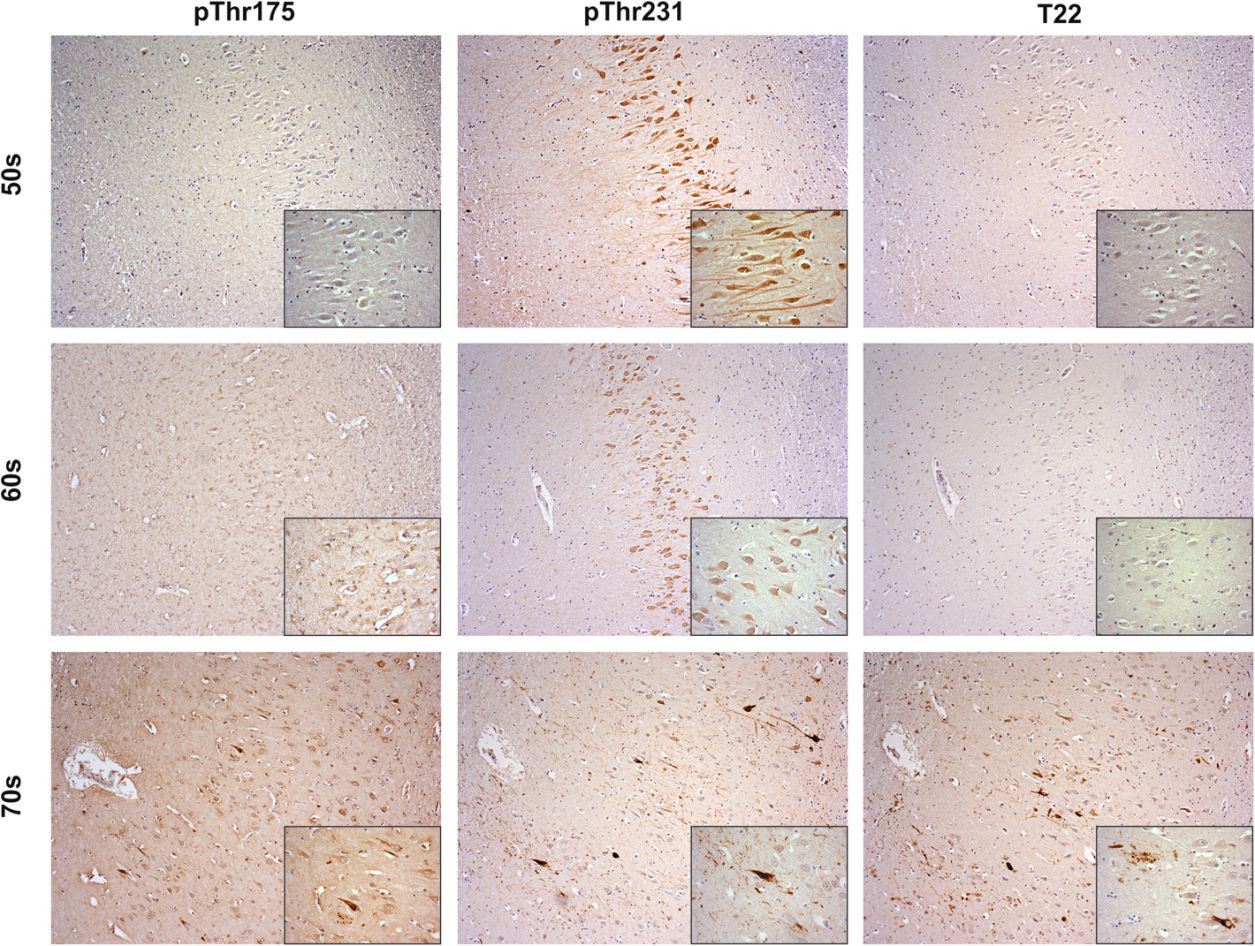
Age dependent tau pathology increases in the hippocampus of controls and is associated with pThr^175^ tau pathology in the 8^th^ decade of life. Large images taken in the CA2 region of the hippocampus at 10× magnification, inset is same region at 40× magnification

Consistent with our previous report, we observed an increase in tau-immunoreactive pathology beginning in the 7^th^ decade of life [22]. In contrast, no immunoreactive inclusions were observed to either the pThr^175^ tau or oligomeric tau (T22 immunoreactivity) in the 6^th^ decade (Fig. 5). pThr^231^ tau immunoreactivity was observed in each of the 6^th^, 7^th^ and 8^th^ decades within the hippocampus. In distinction to the pathological tau deposition observed in both ALSci and the tauopathies, neuronal Thr^231^ tau immunoreactivity was diffuse and localized to otherwise healthy appearing neurons and axonal processes. In the 7^th^ decade, T22 immunoreactive neuronal cytoplasmic inclusions were observed minimally and when present were within the same regions in which we observed punctate pThr^175^ immunoreactivity. All but one case demonstrated pThr^231^ tau immunoreactivity, and importantly this case was negative for all three tau epitopes. In all cases, neuritic pathology was minimal or nonexistent, while neuronal positivity was mainly punctate tau expression.

In the 8^th^ decade, we observed a marked increase in pThr^175^ tau, pThr^231^ tau and T22 immunoreactivity. In this decade, each antibody revealed tau immunoreactive punctate staining of neurons, neurofibrillary tangles, dystrophic neurites, and neuritic plaques. Across all three decades, T22 pathology was present in all cases and in regions where pThr^231^ tau and pThr^175^ tau was present, and was only positive in cases with prominent pThr^175^ tau positive cells and pathology.

## Discussion

In undertaking these studies, we were specifically interested in determining whether the pathogenic phospho-tau species recognized by antibodies against pThr^175^ tau and pThr^231^ tau as well as oligomeric tau (T22) were expressed across a broad range of tauopathies. We were also interested in determining whether these pathological tau species were colocalized in ALS and ALSci. It is known that the phosphorylation of tau protein at Thr^231^ is of both physiological and pathological significance in mediating the dissociation of tau from microtubules [3, 24, 25]. Thr^231^ is phosphorylated by activated GSK3β physiologically and in pathological states [25–29]. We have previously shown that pThr^175^ tau induces GSK3β phosphorylation and that this in turn leads to Thr^231^ tau phosphorylation resulting in tau fibril formation and cell death in vitro [17].

Although the number of cases studied here is limited, the intent was not to undertake a detailed topographic analysis of tau deposition across all tauopathies, but rather to determine whether the proposed pathway of pThr^175^ tau mediated induction of pThr^231^ tau formation with its attendant pathological tau fibril formation (as recognized by T22) was evident. It is noteworthy therefore that we observed that in each tauopathy studied, pThr^175^ tau, pThr^231^ tau and T22 immunoreactivity co-localized to the same inclusion-containing neuronal populations. In each case, neuronal pThr^175^ tau colocalized with pThr^231^ tau. This, paired with the prior identification of pThr^175^ tau in AD brain tissue but not controls [30] and the lack of identified pThr^175^ in fetal tau [15, 16] suggests that pThr^175^ is a key point in pathological tau metabolism, as it is not a physiologically utilized site involved in the regulation of tau function during development or microtubule reorganization. This suggests that the downstream events triggered by pThr^175^ tau, including toxic monomer formation, are common to each of these diseases.

To further assess the pathogenicity of pThr^175^ and pThr^231^, we investigated each epitope in the hippocampus of control cases across three decades of life where tau pathology has been shown to increase with age [22]. We observed no pThr^175^ tau pathology in the 6^th^ decade with minimal immunoreactive neuronal inclusions in the 7^th^ decade. pThr^175^ tau immunoreactivity was most prominent in the 8^th^ decade. In each case in which we observed pThr^175^ tau immunostaining, we also observed T22 immunoreactivity. Similarly, we never observed T22 immunoreactivity in the absence of either pThr^175^ tau or pThr^231^ tau immunoreactivity. In contrast, pThr^231^ tau immunoreactivity was frequently observed in the absence of either pThr^175^ tau or T22 staining in younger individuals and when present, was within healthy appearing neurons and axonal processes. pThr^175^ and T22 did not show pathology in hippocampal regions spared from pThr^231^ pathology, and T22 was only positive in cases showing prominent pThr^175^ pathology.

Glial pathology was recognized to a greater degree by pThr^217^ tau and PHF tau than by the pThr^175^ tau antibody, suggesting that different pathological processes are at play in these cells. This is supported by the lack of identifiable glial pathology by pThr^231^ tau and T22. This paired with the low frequency of pThr^175^ tau glial pathology further strengthens the correlation between pThr^175^ and pThr^231^ in the induction of neuronal pathology and provides evidence that this pair of phosphorylation sites may be exerting specific neuronal toxicity in the disease process across multiple tauopathies.

Although limbic regions universally presented tau pathology, frontal and ACC tau pathology was present mainly in AD, VD, ALSci, FTLD, mDLBD and MSA. This paired with the deeper layer pathology in this region may indicate that tau pathology did not originate here but instead propagated from other regions. If tau originates in limbic structures, propagating along the Papez circuit, it is possible that it would arrive in ACC through thalamic projections to layer IV and V which could act as a hub for propagation to other brain regions such as frontal cortex through this well connected region. Regardless of the induction cause or place, tau protein toxicity is undeniable once initiated [23, 31], and must be considered when attempting to understand the underlying biology of many neurodegenerative diseases. This hypothesis also implies that disease entities such as primary age-related tauopathies (PART) [32] may be in fact not age-related, but neuronal stress related, as increasing age would indicate longer time periods for stresses on neurons to become pathological through stochastic processes [33]. Therefore, tau protein deposition should not be considered a simple function of normal ageing, but ageing should be considered a risk factor for tauopathy among a plethora of neuronal stresses. Of note as well is the frontal involvement in ALSci, which can be concluded is not likely a result of PART, which spares the neocortex by definition [22, 32]. We cannot conclude, however if the layer distribution of tau pathology resembles PART, as this was not described in the consensus report.

## Conclusions

These findings implicate a toxic axis of phosphorylation events beginning with Thr^175^ phosphorylation, dependent on further phosphorylation at Thr^231^, which appears to be neuron specific and which may be common to the tauopathies. The extent to which this process is reflective of not only the presence of a tauopathy, but the disease load as would be encapsulated in the “ABC” score will require much greater case numbers across each disease state. In this paper, we simply raise the critical question that this pathway of pathological tau phosphorylation may be a contributor to neuronal death in these diseases, and thus a point of intervention capable of slowing disease progression resulting from tau protein toxicity.

## Additional files

Additional file 1: Figure S1. Representative PHF tau pathology in each neurodegenerative disease. A) AD hippocampus, B) ALS amygdala, C) ALSci superior frontal cortex, D) ALSci superior frontal cortex astrocytic tau E) CBD entorhinal cortex, F) DLBD amygdala, G) mDLBD entorhinal cortex, H) FTLD entorhinal cortex, I) MSA amygdala, J) PD entorhinal cortex, K) Pick’s amygdala, L) VD anterior cingulate cortex. Nuclear fast red or hematoxylin counterstain used. Original images taken at 100×. (TIF 30305 kb)
Additional file 2: Figure S2. Representative pSer^208,210^ tau pathology in each neurodegenerative disease. A) AD substantia nigra, B) ALS amygdala, C) ALSci entorhinal cortax, D) ALSci ACC neuritic plaque, E) CBD entorhinal cortex, F) DLBD entorhinal cortex, G) mDLBD amygdala, H) FTLD superior frontal cortex, I) MSA amygdala, J) PD entorhinal cortex, K) Pick’s entorhinal cortex, L) VD superior frontal cortex. Nuclear fast red or hematoxylin counterstain used. Original images taken at 100×. (TIF 30111 kb)
Additional file 3: Figure S3. Representative pThr^217^ tau pathology in each neurodegenerative disease. A) AD anterior cingulate cortex, B) ALS entorhinal cortex, C) ALSci hippocampus, D) ALSci superior frontal cortex astrocytic plaque, E) CBD entorhinal cortex, F) DLBD hippocampus, G) mDLBD amygdala, H) FTLD amygdala, I) MSA amygdala, J) PD entorhinal cortex, K) Pick’s entorhinal cortex, L) VD entorhinal cortex. Nuclear fast red or hematoxylin counterstain used. Original images taken at 100×. (TIF 30702 kb)
